# Digital screen time and its effect on preschoolers’ behavior in China: results from a cross-sectional study

**DOI:** 10.1186/s13052-020-0776-x

**Published:** 2020-01-23

**Authors:** Guodong Xie, Qianye Deng, Jing Cao, Qing Chang

**Affiliations:** Pediatric Ward, 7th floor, No. 1 Building, No. 8 People’s Hospital of Wuxi City, No. 1 Guangrui Road, Liangxi District, Wuxi, 214000 Jiangsu China

**Keywords:** Preschoolers, Digital screen time, ADHD, Children, Caution

## Abstract

**Background:**

The aims of the study were to determine the effects of electronic exposure on behaviors of preschoolers, which could provide scientific grounds to the control of digital screen time and usage of electronics.

**Methods:**

Children of 3–6 years of age (*n* = 1897) and their families were included in this study. The daily screen time were recorded for seven days. Children were grouped based on daily screen time of < 60 min or > 60 min. Socio-demographic characteristics of the children were acquired by parental questionnaires. Analyses were made based on the CBCL/1.5–5 results. Chi-square test, t-test and Nonparametric correlation analyses were used to determine the correlation between strength, direction and significance of the relations between the variables. Rates of attention-deficit hyperactivity disorder (ADHD) of children in two groups were compared using χ^2^ test.

**Results:**

Our results indicate that screen time is closely correlated with gender of children, household location, maternal education. We observed that preschoolers with screen time of > 60 min tend to have more behavioral problems than those with screen time of < 60 min (total problem: 35.84 vs. 32.76, *p* = 0.024; externalizing: 11.54 vs. 9.08, *p* = 0.016).

**Conclusion:**

Our study suggests that excessive screen time may be a detrimental factor in the development of preschoolers. Caution should be taken in shortening the screen time of preschoolers.

## Background

The invention of telegraph in the nineteenth century marked a new era of communication and entertainment. Currently, screens which are in form of either smart phones, television, computers, or in theatres, constitute an integral part of daily life. Although the electronics have become essential to all aspects of daily life, children are unavoidably exposed to digital media earlier in life and for longer hours with children in affluent homes with an internet-connected device spending more than 2 h a day on the screens [1]. This time is above the pediatric guideline that recommends less than 1 h per day on screen [2].

There is growing concern that this exposure to electronics could have negative effects on the growth and development of children. Evidences exist showing an association between screen time with obesity, with suggested mechanisms being an increase in the energy intake [3], reduction in the time available for engaging in physical activity [4] or more directly through reduction in metabolic rate [5]. Evidences indicate the link between high screen time to deleterious effects on irritability, negative mood and cognitive and socioemotional development, consequently leading to poor educational performance [6]. These risks are not limited to school-aged children, but also preschoolers of age 2–6.

Another negative impact is on sleep, whereby it has been shown that excessive screen time affects time sleep time and sleep quality and this can consequently result in high blood pressure, low cholesterol levels, cardiovascular diseases, etc. [7]. Other far reaching ramifications include the effects on eyesight, decrease in bone density, tendency to develop depression and suicidal thoughts, difficulty to focus, and attention deficit hyperactivity disorder (ADHD), are possible consequences of excessive screen time [8].

ADHD is a common childhood psychiatric disorder, affecting 5–10% of school-aged children worldwide [9]. Data from population surveys suggest that ADHD is associated with lifestyle related behaviors such as screen time, eating behavior and physical activity with children having ADHD having a higher likelihood of being obese [10, 11]. Contradicting results have previously been obtained that association between screen time and ADHD exists [12]. In a longitudinal study to examine the long term effect of early exposure of television to pre-school children aged between 1 to 3 years, it was shown that an increase of the number of hours that a child watched television at age 1 predicted a 28% increase in attention problem when the child reaches age seven [13]. In other studies, an association between television exposure and ADHD symptoms in children has also been suggested [10, 14]. However, in two other prospective studies, no significant association was found between hours of watching TV and ADHD [15, 16]. There is an urgent need to clarify the relationship between ADHD and screen time in children.

Thus far, studies are more focused on the effects of addictive use of digital media on schoolers, but rarely on preschoolers. In China, there is a profound lack of knowledge of the risk of digital media exposure on preschoolers, yet preschool ages are key period of children in terms of characteristic, physical and intellectual development. Preschoolers are also extremely vulnerable to influence by digital media, and therefore there is an urgent need to elucidate the effects of digital screen time on preschoolers and provide scientific grounds for developing strategies to control screen times of preschoolers.

In the current study, we set out to perform a cross-sectional study to establish if there exists a correlation between behavioral outcomes and screen time of preschoolers. The hypothesis was that screen time of over 60 min had a positive correlation with the development of significant behavioral problems such as ADHD symptoms. We observed that preschoolers with screen time of > 60 min tend to have more behavioral problems, with the risk of behavioral problems being less among females.

## Materials and methods

### Study population and design

We conducted our cross-sectional study in 42 kindergartens with 3842 subjects in No. 8 People’s Hospital of Wuxi City during 2015 to 2018. Children younger than 3 years old and older than 6 years old were excluded. Children born with Autism Spectrum Disorder (ASD) or ADHD symptoms were also excluded. A total of 3742 parent-child dyads participated in our study and the written consent was acquired. Afterwards, a questionnaire was obtained from parents regarding the characteristics of children, e.g. socioeconomics status (SES), ethnicity, etc. In the following week, parents were asked to record meticulously daily on their children in the form of dairy. After the 1-week observation, parents were asked to complete The Child Behavior Checklist (CBCL) preschool version. We performed analyses on the CBCL. All studies were approved by the Ethics Committee of No. 8 People’s Hospital of Wuxi City (#WXDBRMYY524j3). Meanwhile, to obtain potential covariates related to screen-time or children’s behavior, we designed another questionnaire for the parents, which included the question on socioeconomic status, i.e. family income, marital status, education of parents, maternal marital status, physical activity of their child, sleep duration, maternal depression, primary caregivers.

### Recording preschoolers’ screen time

A template, as reported in a previous study, was used to record children’s screen time by their parents [17]. This template utilizes an acceleration meter to record children’s behaviors. In addition to watching TV/DVD, we added use of tablet computers and smartphones, and playing video games to broaden the definition of screen time. The screen time was recorded in minutes and different weights were given to weekdays and weekends to calculate the accumulated screen time. According to the guidelines, preschoolers should not have screen time of more than 60 min, and we therefore grouped the preschoolers based on screen time of less 60 min or over 60 min.

### Evaluation of preschooler behaviors

Parents completed CBCL/1.5–5, which contains 99 items. Specialists analyzed the checklists. Children’s behaviors were divided into 7 categories, including emotionally reactive, anxiousness/depression, aggressive behavior, attention problems, somatic complaints, withdrawn symptom and sleep problems. Another approach of analysis is to divide the behaviors into two broad-band problems, including internalizing problems and externalizing problems. The former includes emotional reaction, anxiousness/depression, somatic complaints and withdrawn symptoms; the latter includes aggressive behavior, attention problems. CBCL also provided five diagnostic and statistical manuals of mental disorders (DSM-5)-oriented syndrome scales, including affective, anxiety, somatic complaints, attention-deficiency/hyperactivity, oppositional defiant, and pervasive developmental scales. Higher score denotes more severe problems.

### Statistical analysis

R was used for statistical analysis. Socio-demographic characteristics were analyzed using chi-square analysis and Student’s t-test. Nonparametric (i.e. Kendall’s tau-b) correlation analyses were used to evaluate the strength, direction and significance of relations between the observed variables. Independent t-test was used to analyze the effect of gender on syndromes derived from CBCL. Multivariate modeling of behavioral outcomes regressed on screen time, with adjustment for important sociodemographic variables, including gender, only childness, household location, maternal education, marital status, family income, and primary caregiver, is accomplished using general linear modeling.

## Results

### Socio-economic status of the subjects

**Fig. 1:**
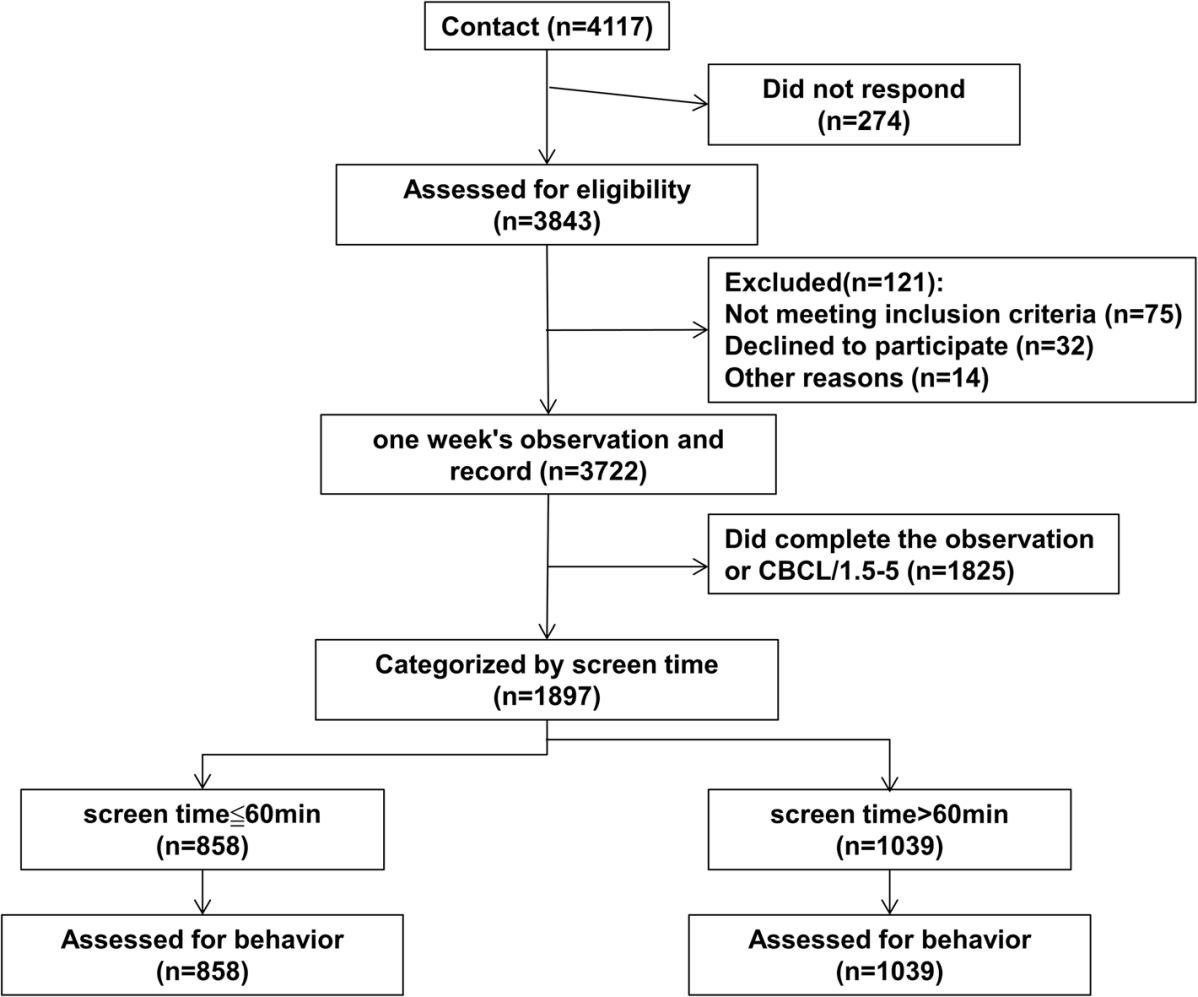
Schematic figure of the experimental design

Design of the experiment is shown in Fig. 1. The socio-demographic characteristic of the subjects that complete the observation of CBCL/1.5–5 (*n* = 1897) is shown in Table 1. The mean screen time is 85 min (SD = 53.47). Thirty percent of the screen time was spent on table/smartphone (25.5 min) and 45% was spent on television viewing (38.25 min), 10% was computer use (8.5) and 15% was video use and others (12.75 min).

**Table 1:** Socio-demographic characteristics of study population

Variables	Screen time		Total (*n* = 1897)	Chi-sq /t test Value	*p*
	≦60 min (*n* = 858)	> 60 min (*n* = 1039)			
Gender, n(%)				9.412	**0.035**
Male	411 (39.7)	623 (60.3)	1034 (54.5)		
Female	447 (51.8)	416 (48.2)	863 (45.5)		
Ethnicity, n(%)				2.864	0.249
Han	796 (44.3)	999 (55.7)	1795 (94.6)		
Minorities	62 (60.8)	40 (39.2)	102 (5.4)		
Age, n(%)				2.386	0.812
3–4 years	192 (31.7)	413 (68.3)	605 (31.9)		
4–5 years	305 (42.8)	408 (57.2)	713 (37.6)		
5–6 years	361 (62.3)	218 (36.7)	579 (30.5)		
The only child, n(%)				3.670	0.625
Yes	473 (56.7)	361 (43.3)	834 (44.0)		
No	385 (36.2)	678 (63.8)	1063 (56.0)		
Household location, n(%)				26.581	**< 0.001**
Rural	210 (26.1)	595 (73.9)	805 (42.4)		
Urban	648 (59.3)	444 (40.7)	1092 (57.6)		
Maternal education, n(%)				10.996	**0.021**
High school or higher	357 (55.8)	283 (44.2)	640 (33.7)		
Secondary	395 (43.3)	517 (56.7)	912 (48.1)		
Primary school or lower	106 (30.7)	239 (69.3)	345 (18.2)		
Marital status, n(%)				10.681	**0.016**
Married	758 (53.4)	66,246.6()	1420 (74.9)		
Divorced	76 (18.1)	348 (82.7)	421 (22.2)		
Widowed	24 (42.9)	32 (57.1)	56 (3.0)		
Family income, n(%)				8.472	**0.034**
< 3000RMB	107 (41.3)	152 (58.7)	259 (13.7)		
3000-5000RMB	348 (40.1)	519 (59.9)	867 (45.7)		
> 5000RMB	403 (52.3)	368 (47.7)	771 (40.6)		
Primary Caregiver, n(%)				20.89	**< 0.001**
Parents	405 (50.8)	393 (49.2)	798 (42.1)		
Grandparents	312 (37.7)	515 (62.3)	827 (43.6)		
Nanny or others	141 (51.8)	131 (48.2)	272 (14.3)		

Notes: Differences were determined by Chi-square tests, except for sleep duration which is by t-test; *p*-values < 0.05 were considered statistically significant, and the data were set in bold

### Correlation between screen time and socio-economic status

The disparity of screen times among preschooler of different gender and age (as indicated by items with *p* < 0.05 in Table 1) prompted us to perform a detailed analysis on the effects of socio-economic status on screen times. As shown in Table 2, screen time was higher in male children (rT = .083, *p* < .01), only-child families (rT = .329, *p* < .0001), rural families (rT = .076 *p* < .05), single-parent families (rT = −.257, *p* < .01), low-income families (rT = .281, *p* < .01), families with lower maternal education (rT = −.272, *p* < .01), grandparent- or baby-site-attended families (rT = −.377, *p* < .01), and children who slept less (rT = − .221, *p* < .01). Some items demonstrate strong positive correlations, while some demonstrated strong negative correlations.

**Table 2:** Observed Zero-Order Correlations Between Observed Variables and screen time (*N* = 1897)

	1	2	3	4	5	6	7	8	9
Gender (male)	–								
Minority ethnicity	.004	–							
Age	.018	.044	–						
The only child	−.423^c^	−.342^b^	.163^a^	–					
Household location	.025	−.162^b^	.036	.013	–				
Marital status	−.017	.123^a^	.040	.051	−.008	–			
Family income	−.078	.178^b^	.005	.224^b^	−.197^b^	.420^b^	–		
Maternal education	.108^a^	.091^a^	.082^a^	.265^b^	−.172^b^	.647^c^	.528^b^	–	
Primary Caregiver	.009	−.039	.005	.151^b^	−.196^b^	.042	−.010	.165^b^	–
Screen time	.083^b^	.007	.055	.329^c^	.076^a^	−.257^b^	.281^b^	−.272^b^	−.377^b^

Notes: Correlation coefficients reflect nonparametric (Kendall’s tau-b) slope estimates

^a^Correlation significant at the .05 level (two-tailed). ^b^Correlation significant at the .01 level (two-tailed). ^c^Correlation significant at the .001 level (two-tailed)

### Effects of screen time on children’s behaviors

To study the effects of screen time on children’s behaviors, the subjects were divided into two groups based on screen time of less or over 60 min in a week. The CBCL/1.5–5 system was used to provide a score on children’s behavior, with a higher score indicating more severe behavioral problems (Table 3). The CBCL is a commonly used method to assess behavioral and psychological problems in children, which reveals internalizing behaviors and externalizing behaviors. It is worth noting that ADHD syndromes are prominently higher in children with > 60 min screen time.

**Table 3:** Mean score comparisons and effect sizes between children categorized by screen time daily on the CBCL/1.5–5 (*N* = 1897)

	Screen time		t value	d′
	≦60 min Mean (SD)	> 60 min Mean (SD)		
Syndromes				
Emotionally Reactive	2.59 (1.76)	3.15 (1.91)	**−4.07**	**0.29**
Anxious/Depressed	2.75 (2.28)	3.46 (2.43)	−0.65	0.04
Somatic Complaints	2.57 (2.48)	2.44 (2.57)	1.04	0.08
Withdrawn	2.51 (2.05)	2.66 (2.19)	−0.90	0.07
Attention Problems	2.37 (1.86)	3.21 (2.11)	**−5.15**	**0.30**
Aggressive Behavior	6.76 (5.43)	7.04 (6.70)	−0.99	0.07
Internalizing	9.58 (7.45)	10.64 (8.47)	**−3.54**	**0.23**
Externalizing	9.08 (6.87)	11.54 (7.51)	**−6.30**	**0.42**
Total Problems	32.76 (19.05)	35.84 (20.43)	**−4.60**	**0.31**
DSM-Oriented Scales				
Affective Problems	2.79 (2.05)	2.65 (1.98)	1.10	0.08
Anxiety Problems	3.98 (2.67)	4.07 (2.95)	−1.02	0.05
Pervasive Developmental Problems	3.54 (3.05)	3.70 (3.17)	−1.15	0.08
ADHA Syndromes	2.97 (2.67)	5.10 (3.75)	**−8.89**	**0.46**
Oppositional Defiant Problems	2.55 (2.40)	3.12 (3.05)	**−7.03**	**0.36**

Note: *p*-values < 0.05 were considered statistically significant, and the data were set in bold

Multivariate modeling of behavioral outcomes regressed on screen time, with adjustment for important sociodemographic variables, including gender, only-childness, household location, maternal education, marital status, family income and primary caregiver is shown in Table 4. We observed that preschooler with screen time of > 60 tend to have more behavioral problems (total problem: *p* = 0.024, externalizing: *p* = 0.016). After regression by screen time, risk of behavioral problem is less among females (total problem: *p* = 0.035; externalizing: *p* = 0.017).

**Table 4:** Effect of Zinc on children’s behavior problems (*N* = 1897)

Variable	Total Problems (R^2^ = 0.12)			Internalizing (R^2^ = 0.07)			Externalizing (R^2^ = 0.14)		
	β (SE)	95% CI	p	β (SE)	95% CI	p	β (SE)	95% CI	p
Screen time									
> 60 min	3.43 (1.33)	0.70–6.16	**0.024**	2.63 (0.31)	−0.71–5.97	0.180	4.04 (0.28)	1.17–6.91	**0.016**
Gender									
Male	–	–	–	–	–	–	–	–	–
Female	−3.07 (1.21)	−8.26–2.12	**0.035**	−0.87 (0.54)	−2.32–0.58	0.894	−4.43 (0.38)	−6.07−− 2.78	**0.017**
The only child									
Yes	–	–	–	–	–	–	–	–	–
No	−4.54 (1.47)	−6.04−−2.04	**0.029**	−3.63 (0.32)	−4.87−− 2.39	**0.015**	−1.63 (0.91)	−2.78−−0.58	0.175
Household									
Rural	–	–	–	–	–	–	–	–	–
Urban	−3.96 (0.61)	−6.58−−1.34	**0.041**	−2.55 (0.83)	−3.03−− 2.07	0.576	−4.05 (2.05)	−7.86−−0.24	**0.046**
Maternal education									
High school or higher	–	–	–	–	–	–	–	–	–
Secondary	1.21 (1.83)	0.45–1.97	0.249	0.72 (0.53)	0.07–1.35	0.594	1.45 (0.37)	−0.05–2.95	0.160
Primary school or lower	2.58 (0.95)	0.71–4.45	**0.042**	0.74 (0.54)	0.06–1.42	0.301	2.79 (0.59)	1.34–4.24	**0.042**
Marital status									
Married	–	–	–	–	–	–	–	–	–
Divorced	3.83 (1.08)	1.36–6.30	**0.045**	5.17 (2.13)	2.63–7.71	**0.022**	4.50 (1.26)	−1.63–10.63	**0.025**
Widowed	3.09 (0.91)	−5.01–11.19	**0.044**	4.07 (1.14)	0.69–7.45	**0.037**	2.90 (0.46)	−0.99–6.79	0.091
Family income									
< 3000RMB	–	–	–	–	–	–	–	–	–
3000-5000RMB	−3.49 (1.18)	−8.35−−1.37	**0.046**	0.33 (0.04)	−2.28−− 1.62	0.098	−0.66 (0.24)	−1.96–0.70	0.150
> 5000RMB	− 2.61 (1.57)	−3.84−−1.38	**0.032**	−0.69 (0.47)	−1.18−− 0.20	0.346	− 1.22 (0.96)	−2.91–0.47	0.079
Primary Caregiver,									
Parents	–	–	–	–	–	–	–	–	–
Grandparents	2.05 (1.37)	1.77–2.33	0.052	1.67 (0.85)	1.17–2.27	0.619	1.42 (0.34)	−2.90–4.32	0.054
Nanny or others	4.41 (0.58)	2.37–6.05	**0.021**	3.27 (0.32)	1.89–4.66	**0.019**	2.66 (0.54)	0.40–5.08	**0.037**

Note: *p*-values < 0.05 were considered statistically significant, and the data were set in bold

## Discussions

While it is increasingly recognized that early and longer exposure to screen has adverse effects on the development of children, scientific evidences supporting this notion is in lack particularly for preschoolers. This study aimed to validate the effect of screen time on behaviors of preschoolers, which could provide guidelines for screen time for preschoolers.

Here we listed a number of factors that may aggravate the correlation between long screen time and behavioral problems (Table 1). The factors that had a significant correlation included gender, household location, maternal education, marital status and primary care giver. Being a boy has a significant correlation with a greater exposure of screen time. This observation is in agreement with other previous studies that boys have a longer screen time compared to girls. Hinkley et al. [18] showed that there was a difference in the correlation of preschoolers’ screen compliance between boys and girls. A recent study by Tamana et al., [19] showed that boys had a higher CBCL externalizing T-score than girls and that boys were more likely to be classified as having clinically significant externalizing behavior problems than girls. Therefore, guidance to reduce preschoolers’ screen use should include sex-specific strategies.

Single parent family was found to also be a significant factor. Research has shown that parents play a critical role in their children’s access to screen time. As the primary caregivers, parents have the chance to establish behavioral control in the home, often in the form of monitoring and rule setting [20]. Research has shown that single parents easily face time constraints and this may limit their ability to monitor or co-participate in their children’s health-related behaviors [21]. Being an only child was also a strong correlator, as the parents of such children tend to give more leeway to child and not easily limit the screen time. It is therefore important that precaution should be given to male, single-parent family and only child, and screen time should be more strictly limited. Children left with nannies as the primary caregivers also had a higher screen time exposure. This can be largely attributed to the fact that nannies would easily leave the children to watch as long as the child remains calm so that they can do other chores. There is therefore need to sensitize the nannies on regulated screen time when they are the primary caregivers.

The consequences of exposing preschoolers to screen time of > 60 min is that they are at higher risk of negative effects on temper, character, and vulnerability to inattention and ADHD symptoms. Previous studies have linked emotional well-being and screen time. Twenge and Campbell [22], in a population based study, showed that high screen time was associated with lower well-being, with the high screen users having twice as many individuals who suffered from anxiety or depression diagnosis. Tong et al., [11] showed that there was an increased risk of obesity in children with ADHD symptoms and this was associated with the overuse of electronic devices, eating while using electronic devices, and delaying bedtimes to snack and use electronic devices. Although previous studies have shown contradicting results in an attempt to examine the association between screen time and ADHD [12], the current study supports the observation that increased screen time has an association with ADHD. It should be noted that there are other negative effects of long digital screen time, such as autism spectrum disorder [23], which further supports the importance of limiting screen time of preschoolers.

One limitation of this study is that we did not take the effects of screen time on physical wellness into consideration, and only focused on behavioral changes. It has been found that hat higher levels of screen time is associated with a variety of health harms for preschool children, with evidence strongest for adiposity, unhealthy diet, depressive symptoms and quality of life [24]. The current study is also a cross-sectional design, and therefore the directional analysis of different parameters is not performed. Further, a more detailed categorization of media devices can be done. In fact, certain educational media exposure has beneficial effects on preschoolers, which has been suggested by many studies [17, 25, 26]. Therefore, a more instrumental guideline should take into account what type, duration and content of screen time would support the development of preschoolers, which warrants further investigation.

## Conclusion

In conclusion, this study suggests that excessive screen time may be a detrimental factor in the development of preschoolers. Therefore, measures need to be put in place to ensure that the screen time is shortened to less than 1 h for preschool children.

## Data Availability

All data generated or analyzed during this study are included in this published article.

## Abbreviation

ADHD: Attention deficit hyperactivity disorder
